# Designing *in vitro* Blood-Brain Barrier Models Reproducing Alterations in Brain Aging

**DOI:** 10.3389/fnagi.2018.00234

**Published:** 2018-08-06

**Authors:** Elena D. Osipova, Yulia K. Komleva, Andrey V. Morgun, Olga L. Lopatina, Yulia A. Panina, Raissa Ya. Olovyannikova, Elizaveta F. Vais, Vladimir V. Salmin, Alla B. Salmina

**Affiliations:** ^1^Department of Biochemistry, Medical, Pharmaceutical & Toxicological Chemistry, Krasnoyarsk State Medical University named after Prof. V.F. Voino-Yasenetsky, Krasnoyarsk, Russia; ^2^Research Institute of Molecular Medicine & Pathobiochemistry, Krasnoyarsk State Medical University named after Prof. V.F. Voino-Yasenetsky, Krasnoyarsk, Russia; ^3^Department of Medical and Biological Physics, Krasnoyarsk State Medical University named after Prof. V.F. Voino-Yasenetsky, Krasnoyarsk, Russia

**Keywords:** blood-brain barrier, neurodegeneration, aging, model *in vitro*, cell senescence

## Abstract

Blood-brain barrier (BBB) modeling *in vitro* is a huge area of research covering study of intercellular communications and development of BBB, establishment of specific properties that provide controlled permeability of the barrier. Current approaches in designing new BBB models include development of new (bio) scaffolds supporting barriergenesis/angiogenesis and BBB integrity; use of methods enabling modulation of BBB permeability; application of modern analytical techniques for screening the transfer of metabolites, bio-macromolecules, selected drug candidates and drug delivery systems; establishment of 3D models; application of microfluidic technologies; reconstruction of microphysiological systems with the barrier constituents. Acceptance of idea that BBB *in vitro* models should resemble real functional activity of the barrier in different periods of ontogenesis and in different (patho) physiological conditions leads to proposal that establishment of BBB *in vitro* model with alterations specific for aging brain is one of current challenges in neurosciences and bioengineering. Vascular dysfunction in the aging brain often associates with leaky BBB, alterations in perivascular microenvironment, neuroinflammation, perturbed neuronal and astroglial activity within the neurovascular unit, impairments in neurogenic niches where microvascular scaffold plays a key regulatory role. The review article is focused on aging-related alterations in BBB and current approaches to development of “aging” BBB models *in vitro*.

## Introduction

Brain aging is one of the most intriguing issues in modern neurobiology, physiology and cell biology. Effects of aging on pivotal brain functions, i.e., cognition and social behavior, are well-known, however, the molecular basis on neurological deficits appearing in the aging brain and showing strong tendency to further redoubling is not fully understood. Numerous experimental and clinical data suggest that the complex of alterations is of great importance for the development of functionally incompetent brain in aged individuals. Such complex includes excessive neuronal death associated with impaired neurogenesis and gray matter shrinking, gliopathy associated with neuroinflammation, excitotoxicity and demyelination, disturbed cerebral microcirculation, imbalanced production of regulatory molecules, altered blood-brain barrier (BBB) permeability and impaired functional brain networks (Peters, 2006; Desai et al., 2010; Antonenko and Flöel, 2014; Bajaj et al., 2017). In this context, age-associated brain disorders might be considered as a particular case of accelerated aging or as an example of disturbed aging process. Also, it is clear that several exogenous factors may affect brain aging either negatively (stress, intoxication etc.) or positively (physical exercise, cognitive training, balanced diet etc.). Most importantly, deciphering molecular mechanisms of their action would help in preventing brain dysfunction or in developing up-to-date therapeutic strategy aimed to control aging process in the brain.

**Figure 1:**
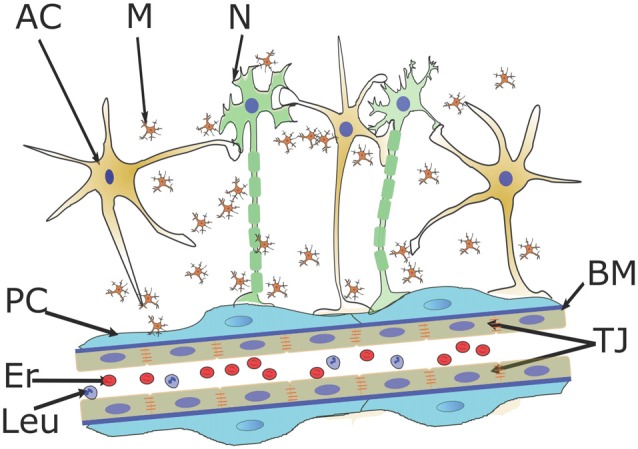
Blood-brain barrier (BBB) structure and intercellular communications within the neurovascular unit (NVU). Brain microvessel endothelial cells (BMECs), pericytes (PC), basement membrane (BM), perivascular astroglial cells (ACs), microglia (M) and neurons (N) form the NVU where endothelial cell layer controls bidirectional transport of macromolecules and fluids, trafficking of immune and progenitor cells to the brain tissue. NVU serves as a platform for neuron-astroglia metabolic coupling, gliovascular control of microcirculation, glia- and pericyte-mediated control of BBB permeability and angiogenesis, vascular support of neurogenesis. Er, erythrocytes; Leu, leukocytes; TJ, tight junctions.

BBB is a highly specialized complex of cells within so-called neurovascular unit (NVU) that are responsible for brain tissue protection, controlled bidirectional transport of fluids, endogenous and exogenous (macro) molecules, immune and progenitor cells trafficking, primarily, at the level of cerebral capillaries. In mammals, BBB/NVU includes cerebral endothelial cells lying on the thin basement membrane (BM), pericytes (PC), perivascular astroglia, neurons and microglia (Abbott and Friedman, 2012; Banks, 2016). A main component of the BBB is a monolayer of brain microvessel endothelial cells (BMECs) that are characterized by high mitochondrial content, low fenestration level, substantial expression of tight junction proteins, small perivascular space, pronounced coverage with astroglial end-feet, and expression of wide spectrum of transporters and receptors (Figure 1). Intercellular communications play prominent role in the regulation of BBB establishment in embryos and in the early neonatal period (barriergenesis), maintenance of BBB structural and functional integrity in the adult brain, in acquisition of barrier properties in newly-formed cerebral vessels in (patho) physiological conditions (reparative angiogenesis, plasticity-associated angiogenesis; Salmina et al., 2015b; Dudvarski Stankovic et al., 2016; Lecuyer et al., 2016; Osipova et al., 2018).

Current interest for molecular mechanisms of BBB development and function is based on real clinical needs. Particularly, overcoming the barrier for the targeted delivery of drugs to the brain tissue, monitoring the barrier status and controlling its permeability in pathological conditions (neuroinflammation, stroke, brain edema), testing new drugs-candidates in *in vitro* and *in silico* BBB models are among top technologies expected to transform the therapy of central nervous system disorders (Albrecht et al., 2016; Zhang Y. Y. et al., 2016; Semyachkina-Glushkovskaya et al., 2017a,b; Bolwerk et al., 2018).

BBB dysfunction in the aging brain relates to various mechanisms, including loss of structural barrier integrity, lower functional coupling of cells contributing to the barrier establishment, PC loss, disturbed activity of BMECs within clonogenic niches (i.e., neurogenic niche, oligovasculogenic niche), altered activity of BBB molecular transporters, and impaired activity of glymphatic system (Zeevi et al., 2010). Thus, simplest characteristic of aging BBB is a loss of structural and functional integrity resulting in pathological permeability and development of abnormal barrier-related events (edema formation, neuroinflammation, insufficient clearance of brain metabolites). Paradoxically, even BBB breakdown associated with enhanced BBB permeability is a key property of aged brain, targeted drug delivery to the brain tissue remains an unresolved problem in gerontology (Erdő et al., 2017). Thus, reconstruction of “aging” phenotype of BBB *in vitro* would be very useful in a huge area of experimental and clinical applications.

## Brief Overview of BBB Models *in Vitro*: Current Opportunities and Challenges

BBB modeling *in vitro* is a rapidly developing area of research which covers study of intercellular communications and development of BBB, establishment of barrier properties pivotal for BBB controlled permeability, development of new scaffolds for the growth of BMECs and application of modern analytical techniques for screening barrier permeability for selected drug candidates and drug delivery systems. In the majority of cases, the following factors are critical for obtaining the fully competent BBB/NVU models *in vitro*. First of all, nature of cellular constituents of the model (mature cells isolated from brain issue, or undifferentiated cells of embryonic origin further subjected to proliferation and desired differentiation *in vitro* or induced pluripotent stem cells (iPSCs)-derived cells) is of great importance. Cells of different origin may have very specific properties in relation to the characteristics of real BBB (i.e., level of fenestration of endothelial cells, perivascular morphology of astroglial cells (ACs), or degree of cell differentiation). Second, establishment of monolayer or multilayer analog of the barrier should be taken into the consideration according to the general task of modeling. As an example, *in vitro* monolayer endothelial barrier might be sufficient for some screening procedures in experimental neuropharmacology but is almost useless for studying barriergenesis or complex intercellular communications within the NVU. Third, still there is no appropriate artificial analog of the BM underlying endothelial cells layer in cerebral capillaries, therefore, production and application of (bio) scaffolds or permeable membranes optimal for endothelial cells functional activity are highly recommended. Fourth, stability of the model and its structural and functional integrity should be achieved and easily maintained *in vitro*. Such barrier properties provide reliable experimental data and guarantee reproducibility of results obtained. Fifth, recent progress in microfabrication technologies allows designing microfluidic models and microphysiological systems that resemble many of the crucial properties of real BBB in the context of blood flow-mediated effects on endothelial cell layer, and finally, a model should allow some desired manipulations with the cells or microenvironment in order to reconstruct conditions typical for different phases of brain development or brain pathologies. As an example, one may manipulate with the expression profile of BBB/NVU cells, may include non-NVU cells or stem cell-derived cells into the model, or expose the model to the action of exogenous physical, chemical or biological factors (Lippmann et al., 2011; Khilazheva et al., 2015; Ruck et al., 2015; Kuvacheva et al., 2016).

In sum, technology of BBB modeling *in vitro* consists of the following steps: (1) *selection* of appropriate cells (primary cultures, cell lines, stem/progenitor cells-derived cells) known as components of NVU/BBB (i.e., endothelial cells, PC, astrocytes etc.) with the special focus on the origin and properties of endothelial cells (i.e., BMECs, human umbilical vein endothelial cells (HUVECs) etc.); (2) *reconstruction* of NVU/BBB microenvironment by seeding the cells in the appropriate medium and in the designed microarchitecture (i.e., 2D model, 3D model, spheroid model, or models with contacting or non-contacting cells in a transwell) on scaffolds supporting their growth and interactions (i.e., gelatin, polylactic acid, biopolymers etc.) in static or microfluidic conditions; (3) *model validation* by the assessment of barrier’s structural and functional integrity and selective transport activity: measurements of transendothelial electric resistance (TEER) which reflects the integrity of endothelial layer and paracellular permeability, analysis of expression of tight junction and adhesion proteins (i.e., zonula occludens 1 (ZO1), junctional adhesion molecule (JAM), occludins, claudins (CLDNs), vascular endothelial cadherins (VE-cadherins) etc.), influx and efflux transporters like glucose transporters (GLUT), monocarboxylate transporters (MCT), P-glycoprotein (Pgp), receptors for advanced glycation end products (RAGE), transferrin receptors; and evaluation of the barrier’s permeability for various molecules and complexes (i.e., liposomes, dextrans, dyes, labeled ligands etc.); and (4) *application of the model* to the given research tasks (i.e., assessment of drug transport or cells trafficking, analysis of intercellular communications or angiogenesis-related events).

**Figure 2:**
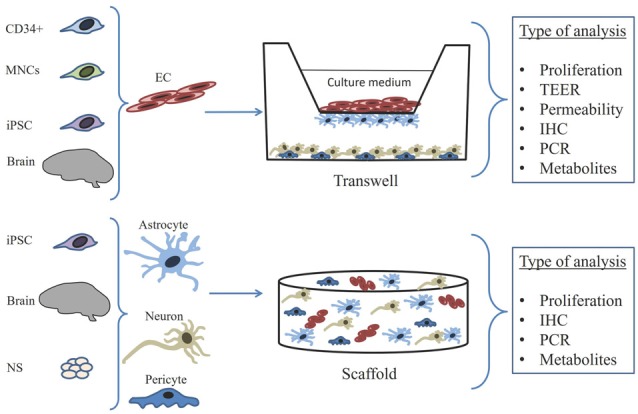
Scheme illustrating general approaches to establishment and validation of BBB *in vitro* models consisted of neurovascular unit cells isolated from the whole brain or cerebral microvessels, or differentiated from stem/progenitor cells, induced pluripotent stem cells (iPSCs) and neurospheres (NS). Three major types of BBB models are shown schematically, including transwell system, 3D model. Right panel shows current opportunities in the assessment of BBB *in vitro* model’s integrity and functional competence. TEER, transendothelial electric resistance; NS, neurosphere; MNCs, bone marrow-derived mononuclear cells; EC, endothelial cells; IHC, immunohistochemistry.

Currently, there are various BBB models *in vitro* that can be classified according to their general properties: (1) monocellular/monolayer models (consisting of BMECs) vs. multicellular/co-culture models (including BMECs, PC, ACs etc.); (2) static models (with stationary extracellular fluid) vs. microfluidic models (with controlled flow of fluid mimicking natural blood flow in cerebral microvessels); (3) brain cell-derived models (i.e., based on BMECs and astrocytes) vs. non brain cell-derived models (i.e., based on HUVEC or other cells lines) according to the origin of cells included into the model; and (4) models obtained from primary cultures vs. those obtained from the established cell lines. Also, BBB models *in vitro* might be restricted to the exact stage of ontogenesis (i.e., BBB model derived from antenatal or neonatal brain cells to study early events in barriergenesis) or to some pathological conditions (i.e., BBB model specific for neurodegeneration or neuroinflammation; Garberg et al., 2005; Helms et al., 2016; Bosworth et al., 2018; Figure 2).

It is obvious that advantages and disadvantages of either BBB *in vitro* model arise from their principal characteristics. For instance, widely-used transwell models allow testing intercellular communications but properties of scaffolds have a special significance, whereas spheroid cultures require no scaffolds but they do not reconstruct natural architecture of the barrier (Ruck et al., 2015), microfluidic models are assumed to be optimal for studying drug pharmacokinetics and allow desired reconfiguration of fluid flow within the device but they are rather expensive and possess high technical requirements (Bonakdar et al., 2017).

Diversity of BBB models allows performing pre-clinical screening of drug candidate and studying barriergenesis, transport machinery, and pathological conditions associated with the dysfunction of the NVU (Stanimirovic et al., 2015). Even “ideal” BBB models *in vitro* do not exist yet, however, several attempts to produce renewable, controlled, scalable and fully functional BBB models have been realized. As an example, BBB models based on cells originated from embryonic or adult stem cells became to be rather popular. In such protocol, BMECs or astrocytes are differentiated from immature stem cells, then they are phenotyped to get the populations with characteristics of desired cell type, and are further co-cultured under appropriate conditions (Malinovskaya et al., 2016). Very recent achievements in modeling BBB *in vitro* have been connected with the application of human iPSCs-based protocols for getting BMECs, PC and other perivascular cells representing high degree of similarity to natural BBB (Bosworth et al., 2018; Ribecco-Lutkiewicz et al., 2018). One can expect that further progress in the establishment of BBB models *in vitro* might be done with the application of gene-targeted strategies to modify expression of proteins directly involved into the regulation of BBB integrity. For example, Cre-Lox protocol has been successfully used for the disruption of Smad proteins acting downstream of TGF-β receptors in cerebral endothelial cells (Li et al., 2011) or for the inactivation of β-catenins involved in the stabilization of endothelial tight junctions (Liebner et al., 2008). Therefore, these protocols might be further applied for creating novel BBB models with target modification of barrier properties *in vitro*. Analogous prospects arise from the application of precise gene editing technologies, i.e., clustered regularly interspaced short palindromic repeats (CRISPR)/Cas9-based protocol enabling manipulation with functional properties of BBB/NVU cells (Zhou et al., 2018), or optogenetic approaches either *in vivo* and *in vitro*.

Development of microphysiological systems containing BBB *in vitro* model opens new stage in the establishment of functionally competent BBB *in vitro*. Combination of microfluidic technology and establishment of multi-tissue ensembles (i.e., neurons, liver cells, placenta cells etc.) allows studying complex inter-tissue communications relevant for various (patho) physiological conditions, performing drug screening, and creating novel test-systems for the development of drug delivery protocols (Brown et al., 2015; Phan et al., 2017; Edington et al., 2018).

## Properties of BBB/NVU Constituents in Aging Brain

Phenomenon of brain aging is in the focus of neurobiologists and neurologists. Aging is a risk factor for specific types of neurodegeneration, i.e., in Alzheimer’s disease (Guerreiro and Bras, 2015), however, pathological brain aging itself represents aggravated or completely altered program of natural senescence caused by exogenous and endogenous factors. It is interesting, that regardless type of aging, neuroplasticity reserve seems to be preserved in various brain areas (Cotelli et al., 2012), even it is still debated whether pathological neurodegeneration is a type of excessive aging process (Ghosh et al., 2011). Moreover, some other factors could affect brain physiological and pathological aging. It was found that volumes of subcortical structures were much more preserved in physiological aging women comparing to men (Kiraly et al., 2016), thus suggesting that—at least in humans—male brains might be more susceptible to aging. However, some contradictory data exist as well (Greenberg et al., 2008). In pathological aging, i.e., in Alzheimer’s disease development, female brains seem to be more susceptible to acquiring senescent phenotype (Zhao et al., 2016), presumably, due to specific features of metabolism or because of alterations in signaling pathways coupled to receptors of growth factors and neurosteroids.

Anyway, chronic neurodegeneration in aging brain is always accompanied by the phenomenon of vascular aging (Donato et al., 2015). It is a complex gradual process resulting in NVU dysfunction, impairment of neurogenesis and angiogenesis. As an example, in retina, progressive vascular damage in aging rats is a multistep process consisting of thickening of the basal capillary membrane, disorganization of PC cytoskeleton at the earliest stage followed by microvessel remodeling and angiogenesis at the latter stage (Hughes et al., 2006). Endothelial cells, PC, and perivascular astroglia are implicated in the pathogenesis of cerebral vascular aging. Interestingly enough, brain vascular aging and endothelial leakage in humans may start in hippocampal microvessels, thereby preceding hippocampal atrophy and cognitive decline (Montagne et al., 2015). It should be also noted that hippocampus demonstrates the highest levels of amyloid-induced hyper vascularization and leaky BBB in Alzheimer’s type of neurodegeneration (Desai et al., 2009; Biron et al., 2011), suggesting that accelerated cerebral vascular aging and neurodegeneration-mediated angiopathy could have causative relationship with the activity of adult neurogenic niches responsible for hippocampus-supported learning and memory: active neurogenic events correspond to the sites of elevated BBB permeability (Lin et al., 2015; Pozhilenkova et al., 2017). As a result of endothelial, PC and astroglial impairments, brain aging is always associated with reduced cerebral capillary blood flow and altered gliovascular local control of microcirculation (Desjardins et al., 2014), BBB breakdown (Elahy et al., 2015), and disturbed synaptic plasticity evident as long-term potentiation (LTP) deficits in brain regions with pathological BBB permeability (Blau et al., 2012).

*Endothelial dysfunction* is a hallmark of normal aging. Senescent endothelial cells are characterized by higher susceptibility to oxidative stress, reduced proliferation and sensitivity to the action of pro-angiogenic factors, excessive cell death (apoptosis and autophagy), compromised ability to prevent blood coagulation events, limited availability and impaired response to vasodilating factors and propensity to support chronic inflammatory process.

BMECs have some important characteristics that make them dramatically susceptible to the above-mentioned processes, particularly, they possess high number of mitochondria (Kluge et al., 2013) and demonstrate strong intercellular coupling provided by tight junctions, adherence junctions and connexin channels (De Bock et al., 2011; Luissint et al., 2012; Dejana and Orsenigo, 2013). As an example, oxidative stress is a well-known marker of aging, whereas oxidative stress-induced thiol oxidation, phosphorylation, nitration and carbonylation of tight junction proteins result in BBB breakdown (Rao, 2008; Enciu et al., 2013). These mechanisms have been shown in D-galactose-induced mouse brain aging (Lei et al., 2013) and were implicated in obesity-promoted cerebrovascular aging in mice (Tucsek et al., 2014). In aged mice, deficit in tight junction coupling is accompanied by leaky BBB and neuroinflammatory events; the latter is not caused by significant leukocytes recruitment into the brain tissue (Elahy et al., 2015).

**Figure 3:**
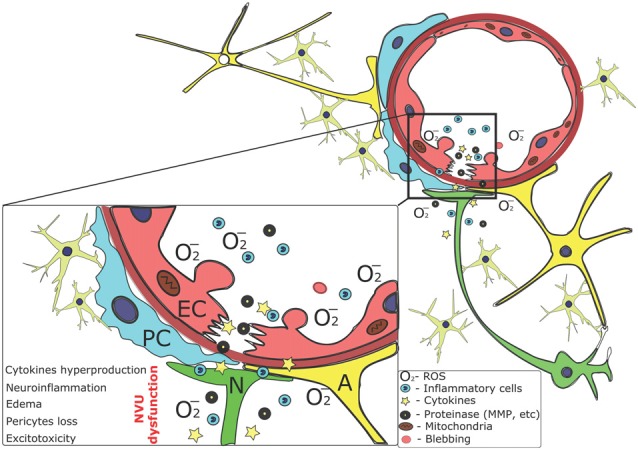
Senescence-associated changes contribute to BBB/NVU impairment in aging. BMECs and perivascular cells serve as a source of cytokines, reactive oxygen species (ROS) and proteases affecting BBB permeability, energy production and metabolism in the barrier cells as well as excitability and synaptic plasticity of neighboring neurons. These events lead to progressive loss of barrier properties, metabolic alterations in affected cells (i.e., ATP and nicotinamide adenine dinucleotide (NAD^+^) depletion), initiation and progression of apoptosis or autophagy, and development of neuroinflammation. As a result, vicious circle is established leading to further changes in BBB structural and functional integrity.

Senescent cells are usually characterized by overproduction of cytokines and proteases, excessive DNA damage response, mitochondrial dysfunction and reactive oxygen species (ROS) generation. All these mechanisms are tightly coupled and potentiate each other. As an example, excessive production of ROS supports development of neuroinflammatory response (El Assar et al., 2013) culminating in the establishment of so-called senescence-associated secretory phenotype (SASP) evident in endothelial and perivascular cells (Chen et al., 2002). Endothelial oxidative stress is associated with prominent activation of poly (ADP-ribose) polymerase needed for DNA repair, thereby resulting in nicotinamide adenine dinucleotide + (NAD^+^) depletion and endothelial cell death (Pacher et al., 2002). Additionally, in the aged brain, altered processing of amyloid precursor protein in endothelial cells leads to amyloid beta (Aβ) accumulation and its deposition in cerebral microvessels (Muche et al., 2017). Progressive accumulation of Aβ in brain microvessels results in the development of cerebral amyloid angiopathy (CAA) and supports persistence of SASP in NVU cells. All these events are always accompanied by mitochondrial dysfunction, insufficient ATP production, accelerated mitophagy and aberrant mitochondrial biogenesis in BMECs, PC and perivascular glial cells (Caja and Enríquez, 2017; Karnewar et al., 2018). Finally, SASP-associated matrix metalloproteinase and cytokines release dramatically disrupts endothelial monolayer integrity and ultimately leads to BBB hyperpermeability, microvessel remodeling and loss of neurovascular control (Goligorsky and Hirschi, 2016; Figure 3).

In cerebral endothelium, aging-related events also include elevated VEGF signaling and lowered NO availability in senescent BMECs resulting in vessels hyperpermeability and hypervascularity (Oakley and Tharakan, 2014). Whether or not VEGF action in the aging brain corresponds to the altered expression of its alternative isoforms that may either increase or decrease BBB permeability, support or suppress angiogenesis (Woolard et al., 2009) remains to be evaluated. Besides, aging affects activity of several transporters expressed in BMECs. Particularly, glucose uptake via GLUT is reduced in the aging rat microvessels (Mooradian et al., 1991), thereby suggesting functional link between aging-associated cerebral glucose hypometabolism and BBB impairments. Aβ clearance through BBB was found to be altered in aging rats due to abnormal expression of low-density lipoprotein receptor-related protein-1 (LRP-1) and Pgp in BMECs (Silverberg et al., 2010). Aberrant expression of Pgp in aging human BBB results in disturbed cerebral pharmacokinetics of several drugs known as Pgp substrates (van Assema et al., 2012). Thus, aging-affected BMECs are characterized by progressive loss of permeability controlling mechanisms resulting in deregulated cerebral metabolism of endogenous molecules and xenobiotics.

*PC dysfunction and loss* are often seen in chronic neurodegeneration and, particularly, in aging. Age-dependent vascular damage in pericyte-deficient mice preceded chronic neurodegeneration and cognitive decline (Bell et al., 2010). Expression of senescence markers in brain PC—β-galactosidase activity and p16 (INK4a)—was associated with compromised BBB integrity in senescence-prone mice (Yamazaki et al., 2016). In the rat frontal cortex and hippocampus, age-related changes in PC microstructure include elevation of mitochondria number, and this phenomenon is much more prominent comparing to BMECs (Hicks et al., 1983). Thus, loss of PC coverage could be partially compensated by stimulated metabolic activity in remaining cells. Indeed, even excessive PC loss can be effectively counteracted in the brain without evident changes in BBB permeability in mice (Villaseñor et al., 2017). PC always show prominent structural plasticity and their distal processes are rather dynamic (Berthiaume et al., 2018) but whether such properties are maintained in normal aging of BBB remains to be clarified. In sum, aging-related changes in cerebral PC mainly caused by their excessive cell death and microstructural rearrangements in the remaining cells might be partially compensated at the earliest stages of BBB breakdown.

*Astroglia is affected in aging* because of oxidative stress and development of local inflammation. As a result, neuron-astrocyte metabolic coupling and astrocyte-endothelial interactions are impaired, thereby leading to aberrant neuronal activity and BBB breakdown in a region-specific manner (Rodríguez et al., 2014). Aging-associated progressive telomere shortening and development of SASP occur in astroglial and endothelial cells (Bitto et al., 2010; Chinta et al., 2013; Morgan et al., 2018), thus contributing to local production of pro-inflammatory cytokines. Besides that, astrocytes in the aging mouse brain are very sensitive to the stimulatory action of activated microglia (Clarke et al., 2018), thereby contributing to the establishment of pro-inflammatory microenvironment in the perivascular area. In aging mice, alterations in paravascular drainage mechanisms provided by glymphatic system were associated with abnormal AQP4 polarization, thereby suggesting aberrant astroglial activity (Kress et al., 2014). Recent transcriptomic analysis of multiple mice brain regions showed that aging astrocytes up-regulate genes encoding proteins that are required for synaptic remodeling and astroglial activation (Boisvert et al., 2018). Moreover, senescent mouse astrocytes demonstrate higher ability to contribute to synapse elimination and show clear brain regional specialization (Boisvert et al., 2018). However, it should be taken into consideration that there is a shortage of data obtained precisely in perivascular astroglia in aging brain, therefore, the question exists whether the observed aging-associated changes in astrocytes could be safely extrapolated on particular NVU ACs whose end-feet are tightly connected with BMECs.

Astrocytes are well-known producers of lactate in active brain regions, and this metabolite could be used by neuronal cells to support their extremely high energetic needs, or could be transported and/or utilized by BMECs to control their mitochondrial activity and angiogenesis-related events (Salmina et al., 2015b). In aging, glycolytic activity of astrocytes may be dramatically compromised: aging ACs show reduction of glycolysis, stimulation of mitochondrial biogenesis and activation of mitochondrial ATP production leading to impaired neuron-astrocyte metabolic coupling and local glucose hypometabolism (Jiang and Cadenas, 2014). These events, probably, result from the establishment of local pro-inflammatory microenvironment within the NVU, since inflammation-affected cells usually activate mitochondrial biogenesis in order to compensate for mitochondrial damage (Piantadosi and Suliman, 2012). Such mechanism could be linked to mitophagy-associated activation of NLRP3 inflammasomes whose activity results in interleukins (IL-1β, IL-18, IL-33) release and inhibition of glycolysis (Tschopp, 2011). Elevated levels of brain lactate is a hallmark of aging-related neurodegeneration, particularly, at the presymptomatic stage in mice (Ross et al., 2010), so, exact molecular mechanisms of deregulated lactate production and transport in the aging brain remain to be assessed. Thus, aging results in astroglial activation and, presumably, impairment of astroglia-supported intercellular communications within the NVU with long-lasting effects on BBB permeability and angiogenesis.

*Microglial effects on BBB in aging* is poorly understood. Actually, microglial cells are not considered as an obligatory component of the NVU, however, numerous studies reveal prominent role of activated microglia in controlling BBB permeability due to secretion of cytokines, chemokines, ROS and several metabolites (da Fonseca et al., 2014; Dudvarski Stankovic et al., 2016; Osipova et al., 2018). Microglia may directly interact with brain microvessels, particularly, with endothelial tip cells governing angiogenesis, thus suggesting the regulatory role of activated microglial cells in cerebral barriergenesis and angiogenesis. Microglia-released interleukins, TNF and ROS potentiate BBB breakdown and elevate paracellular permeability of the barrier, thereby resulting in edema formation and leukocytes trafficking in the affected brain regions. So, microglial activity in a close vicinity to the BBB results in promotion of angiogenesis (hypervascularity) and elevated permeability of newly formed microvessels. It may have a relation to the appearance of numerous microvessels with leaky BBB in Alzheimer’s disease manifested by pronounced neuroinflammation in mice (Biron et al., 2011) as well as to the delayed angiogenesis and impaired microvessel permeability in post-stroke aged rats and humans (Buga et al., 2014).

Age-dependent activation of microglia is a widely-recognized phenomenon (Spittau, 2017), therefore, it is obvious that aging-associated alterations in BBB permeability could be partially caused by microglial cells. One can describe this mechanism as an example of low-grade chronic neuroinflammation with all the corresponding attributes: microglial cytotoxicity, complement activation, phagocytosis, development of cytokine storm, excessive clearance of cellular debris in the loci of excessive apoptosis, microglial M2 → M1 polarization with the corresponding changes in cell metabolism (Chan-Ling et al., 2007; Cerbai et al., 2012). However, very recent data suggest that in addition to cytotoxic and pro-inflammatory phenotype, cortical microglial cells in aged mice may also acquire immunotolerogenic properties (Zöller et al., 2018). In general, priming and even uncontrolled activation of microglia correspond to the progression of brain aging whereas severity of such changes associates with neurological deficits and behavioral abnormalities (Norden and Godbout, 2013).

*BM contributes a lot* to the functional integrity of the BBB, and its role might be compromised in aging, i.e., due to elevated release of MMP-2 and MMP-9 from activated ACs, microglia or PC. MMPs degrade BM proteins, thereby contributing to BBB breakdown (Lee et al., 2012), but they also important for microvessel remodeling and neoangiogenesis. Some data suggest that aging and Alzheimer’s type of neurodegeneration might be associated with significant thickening of the barrier BM (Marques et al., 2013; Yamazaki and Kanekiyo, 2017), but whether it is important for BBB dysfunction seen in the aged brain is not clear yet.

## Mechanisms and Markers of Cell Senescence: Applications for NVU Study in BBB Models *in Vitro*

Probably, the most reliable way to get an NVU/BBB model resembling properties of aging brain tissue might be in obtaining of cells from the aged animal followed by their growth *in vitro* within the multi-cellular ensemble (endothelial cells and perivascular cells) mimicking NVU microenvironment. However, this approach is difficult to execute because proliferation and establishment of appropriate and functionally competent intercellular contacts are very limited in the culture of cells isolated from the aged brain tissue (Salmin et al., 2017). But very recent findings suggest that neurogenic potential of cells in physiological aging might be well preserved (Boldrini et al., 2018). Therefore, aged brain-derived stem and progenitor cells might be considered as a potential source of NVU cells. Another approach is based on the induction of senescence-specific properties in NVU cells obtained at much earlier stages of brain development. In this case, appropriate protocols for inducing and detecting senescence phenotype in cells within the NVU/BBB models *in vitro* should be validated and applied.

*Proinflammatory changes in the microenvironment of senescent cells*. At the cellular level, senescence is characterized by cessation of cell proliferation and establishment of so-called SASP resulting in release of proinflammatory cytokines, chemokines growth factors, MMPs and other proteases, serpins, soluble or shed receptors and their ligands, membrane-derived microvesicles into the extracellular space in order to create microenvironment optimal for senescent cell (Coppé et al., 2010; Robbins, 2017). Recently, cellular senescence has been recognized as a cause of chronic neurodegeneration, i.e., in Alzheimer’s disease, linking neuroinflammation to non-reversible cell aging and accumulation of non-repairable alterations. Moreover, some data suggest that aging is also accompanied by an increase in the number of SASP-expressing senescent cells of non-neuronal origin (i.e., astrocytes, microglia, endothelial cells, neural stem cells) in the brain (Chinta et al., 2015). Therefore, it could be proposed that senescence of non-neuronal cells in the aging brain could contribute to progressive impairment of BBB (aging endothelial cells), insufficient myelination (aging oligodendrocytes), reduced neurogenesis (aging neural stem cells) and development of sterile neuroinflammation (aging astroglial and microglial cells).

Besides, another function of SASP induction might be found in the promotion of cell plasticity and stemness as it was shown in the skin: keratinocytes exposed to the SASP initially elevate expression of stem cell markers and regenerative capacity *in vivo* followed by the cell cycle arrest (Ritschka et al., 2017). Whether or not such mechanism is operative in the brain, particularly, in neurogenic niches with prominent vascular scaffold architecture, remains to be evaluated, but several attempts to produce neuropinflammation-specific models of BBB have been undertaken. As an example, endothelial monolayers arranged within tube-like structures on a microfluidic platform demonstrate SASP-like response to tumor necrosis factor-alpha (TNF-α) action *in vitro* (manifested by the production of 29 cytokines, chemokines and growth factors) and reproduce some properties of inflammation-induced BBB leakage (Cho et al., 2015). Therefore, monolayer BBB models could be used for studying SASP-related changes affecting barrier integrity. The same effect could be achieved by stimulating secretory activity of perivascular astroglia which leads to enhanced release of cytokines and chemokines, thereby supporting its paracrine action at BMECs (Osipova et al., 2018). Particularly, being stimulated with the mixture of IL-1β and TNFα, astrocytes *in vitro* produce upto 30 cytokines, chemokines and soluble mediators, such as complement, growth factors, adhesion molecules, serpins, etc. in a time-dependent manner (Choi et al., 2014). If BBB model is reconstituted consequently with different cell types, preliminary culture of astrocytes with IL-1β and TNF-α would produce SASP-resembling conditions in 24 h after treatment in non-toxic concentrations. Another protocol is based on genetic manipulation with signaling molecules upstream the pro-inflammatory cytokines secretion. Particularly, elevated expression of nuclear factor-kappa B (NF-κB) in PC leads to release of monocyte chemoattractant protein-1 (MCP-1) and IL-8 that are able to stimulate endothelial cell proliferation in skeletal muscle (LaBarbera et al., 2015). Thus, the same approach could be tested in BBB models *in vitro*. In sum, detecting changes in cell secretome specific for SASP is a good marker of cellular senescence, whereas targeting SASP is recognized as an approach to eliminate senescent cells and to prevent aging (Watanabe et al., 2017). Induction of SASP phenotype in BMECs, PC or astroglia is a tool for modeling BBB with the properties specific for aging brain.

*Inflammasome activation and insulin resistance (IR) in senescent cells*. Activation of cell senescence program is always associated with inflammasome induction, particularly, in endothelial cells of large vessels where ROS-driven expression of cryopyrin, NOD-like receptor 3 (NLRP3) inflammasomes well explains elevated release of major pro-inflammatory cytokine IL-1β and acquisition of senescent phenotype (Yin et al., 2017). The same is true for the development of peripheral IR characteristic for so-called inflamm-aging (Bauernfeind et al., 2016). Expression of NLRP3 inflammasomes accompanied by increased expression of MMP-2 and reduced TEER was registered in the BBB *in vitro* model exposed to poly(I:C; Małecki et al., 2017), but whether such approach might be useful in establishing BBB model specific for the aging brain remains unclear.

Brain cells are equipped with various components of insulin signaling machinery. Alzheimer’s type of neurodegeneration is believed to be a particular case of IR and impaired glucose tolerance in the brain tissue (Thambisetty et al., 2013). IR results in aberrant transport and reception of insulin in the brain tissue, thereby contributing to abnormal processing of amyloid precursor protein and excessive Aβ accumulation, altered metabolism of glucose and secondary changes in lactate-driven mechanisms (neuron-astrocyte metabolic coupling, angiogenesis; Willette et al., 2015; Neth and Craft, 2017). In humans, brain aging is associated with progressive loss of insulin receptors (Frölich et al., 1998), and very recent studies suggest that vulnerability of different brain regions to Aβ toxicity corresponds to their sensitivity to local insulin action (Mullins et al., 2017).

Link of cellular senescence and IR has been demonstrated in several cell types, i.e in hepatocytes (Aravinthan et al., 2015), cardiomyocytes (Boudina, 2013). Thus, taken into the consideration that aging is often accompanied by IR, and it is true for brain (Baranowska-Bik and Bik, 2017), one may assume that induction of IR in NVU cells could make them more sensitive to the action of aging-promoting factors *in vitro*. It should be noted that IR prevents effective repair of endothelial layer and suppresses reparative angiogenesis, thereby promoting vascular aging (Avogaro et al., 2013). Thus, disruption of insulin sensing and signaling in NVU cells would help achieving senescence-prone phenotype for study BBB breakdown in aging brain.

*Depleted NAD^+^ levels in senescent cells*. Glucose intolerance in microvessel endothelial cells results in altered glucose uptake and acceleration of their senescence due to suppression of activity of NAD^+^-dependent histone deacetylases sirtuins (SIRTs; Mortuza et al., 2013). Interesting to note, that elevation of NAD^+^ levels in endothelial cells by up-regulating nicotinamide phosphoribosyltransferase (Nampt) delays acquiring the senescence phenotype and promotes angiogenesis (Borradaile and Pickering, 2009). Thus, manipulating NAD^+^ bioavailability, i.e., via NAD^+^-glycohydrolases CD38 or CD157, might be a way to change cell’s sensitivity to the action of aging-promoting factors *in vitro* (Chini, 2009; Camacho-Pereira et al., 2016; Chini et al., 2018), whereas supply of nicotinamide riboside as NAD^+^ precursor would efficiently elevate intracellular NAD^+^ levels (Dellinger et al., 2017) and delay cellular senescence (Zhang H. et al., 2016). CD38 and CD157 serve as receptors and enzymes with NAD^+^-converting activity that have been initially recognized as important regulators of immune cells functional activity (Malavasi et al., 2006). Later, it was confirmed that both the enzymes are widely expressed in the brain being predominantly found in neurons, astrocytes, and microglia (Ceni et al., 2003) where they take part in the action of neurotransmitters, coordinate neuron-astrocyte metabolic coupling, regulate local immune response. In particular regions of the brain (hypothalamic area and pituitary) CD38 controls NAD^+^ metabolism and neurosecretory activity required for oxytocin secretion which regulates various neurobehavioral responses, i.e., interpersonal communications, parental behavior, decision making (Lopatina et al., 2011, 2012; Higashida et al., 2012; Salmina et al., 2012; Akther et al., 2013). Expression of CD38 and CD157 is controlled by pro-inflammatory cytokines, retinoic acid, cell proliferation or differentiations status, and availability of their own ligand NAD^+^ (Malavasi et al., 2008). In endothelial cells, inhibition of CD38 preserves nitric oxide (NO) synthase activity and NO generation, whereas activation of CD38 leads to NADPH depletion, thereby contributing to endothelial dysfunction in the heart (Reyes et al., 2015). Presumably, analogous mechanisms might be active in BMECs since production of NO is greatly compromised in cerebral microvessels in chronic neurodegeneration (Salmina et al., 2015a). CD38 is also expressed in retinal PC being up-regulated by pro-inflammatory cytokines, whereas application of anti-CD38 antibodies resulted in PC injury (Li et al., 2012). Very recent data revealed expression of CD157 in endothelial cells and it function as an angiogenesis-regulating molecule in various tissues, including brain (Wakabayashi et al., 2018). It is tempting to speculate that aberrant expression of CD38 and CD157 in BMECs and cerebral PC might affect NAD^+^ levels and acquisition of senescence phenotype in BBB *in vitro* models.

Oxidative stress is another mechanism causing cell senescence *in vitro*. Partially it is explained by oxidant-induced acute depletion of intracellular NAD^+^ pool due to excessive activation of poly(ADP-ribose)polymerase required for efficient DNA repair. Thus, it is not surprising that one of the methods to induce cell senescence in BBB models *in vitro* is application of oxidants like H_2_O_2_. However, efficacy of H_2_O_2_ may differ depending on cell types and previous passages of cells (particularly, endothelial cells) that affect cell cycle and their sensitivity to the action of genotoxic agents (Yamazaki et al., 2016). However, this approach might be very effective in getting phenotypic changes characteristic for aging BBB.

Glycolysis provides partial regeneration of NAD^+^ pool in the cells, therefore glycolytic activity of NVU cells would have an impact on their aging dynamics. Metabolic profiling of senescent fibroblasts has shown elevated activity of glycolysis that can be explained by mitochondrial dysfunction in aging cells (James et al., 2015). Similar effect has been observed in senescent astrocytes where up-regulation of glycolysis corresponded to the degree of mitochondrial overactivation, ROS production, and inflammatory response of astroglia (Cohen et al., 2017). Thus, elevated glycolytic flux might be a part of cell protective mechanism to prevent NAD^+^ depletion and to delay cell aging.

Recently, we found that activation of GPR81 lactate receptors in BMECs *in vitro* resulted in mitochondrial biogenesis and was accompanied by reduced expression of MCT-1 and CD147 (Khilazheva et al., 2017). Mitochondrial biogenesis is driven by DNA damage in senescent cells and results in elevated production of ROS and cell cycle arrest (Correia-Melo et al., 2016). Thus, enhanced rate of glycolysis in senescent cells could lead to massive efflux of lactate to the extracellular space where it acts at GPR81 receptors and stimulates mitochondrial biogenesis in BMECs. The latter results in excessive production of ROS, DNA damage, and induction of DNA-damage response (DDR) which is a hallmark of aging cells.

*DDR in senescent cells*. One of experimental approaches to induce senescence of cultured cells is based on their exposure to gamma-irradiation which can induce reparable or irreparable DNA damage (James et al., 2015). This method is effective since another important feature of senescent cells is an induction of DDR which is a prerequisite for SASP and is triggered by genomic lesions. It is generally accepted that senescent cells accumulate the phosphorylated form of histone H2AX (γ-H2AX) which marks sites of DNA double strand breaks (DSBs) and is required for maintaining genome integrity (Turinetto and Giachino, 2015). Telomere shortening seen in senescent cells leads to the loss of telomere-bound inhibitors of ATM and other kinases involved in H2AX phosphorylation, therefore, DDR is spontaneously activated (so-called replicative senescence induced by telomere attrition; d’Adda di Fagagna, 2008; Maicher et al., 2012).

In the brain, accumulation of γ-H2AX mainly occurs in neurons and glial cells, but some cerebral endothelial cells demonstrate presence of this marker as well (Barral et al., 2014). Hippocampal astrocytes show high levels of γ-H2AX expression in Alzheimer’s type of neurodegeneration (Myung et al., 2008), endothelial cells need in γ-H2AX for their proliferation in hypoxia-driven neoangiogenesis (Chavakis et al., 2009). H2AX^−/–^ mice demonstrate impaired endothelial cell proliferation (but not pericyte dysfunction) associated with reduced angiogenesis (Economopoulou et al., 2009). In neurons, phosphorylation of H2AX with ATM and DNA-PK occurs in physiological brain activity: glutamatergic stimulation of neurons or exposure to novel environment result in topoisomerase II-dependent DSBs and appearance of γ-H2AX witnessing immediate early genes expression (i.e., c-fos, npas4, egr1) in activated neuronal cells (Suberbielle et al., 2013; Madabhushi et al., 2015). Thus, phosphorylated H2AX might be a good marker of DSBs and/or telomere shortening but not a specific marker of cellular senescence due to its presence in functionally active non-damages cells (Bernadotte et al., 2016). However, location of γ-H2AX in genome could be helpful in discriminating exogenous and endogenous mechanisms of its induction (i.e., ionizing radiation and activation of transcription, respectively). For example, sub-telomeres are less responsive to external DNA damage than to endogenous stress (Seo et al., 2012).

In sum, various experimental approaches should be considered when BBB model is established *in vitro* for studying numerous functional and morphological alterations in aging brain. Manipulations with the cell expression profiles and activity of senescence-associated molecular machinery within NVU/BBB would allow designing and development of BBB *in vitro* models resembling many of aging-coupled phenomena. Table 1 summarizes up-to-date data on main molecular mechanisms and markers of aging-associated alterations in NVU cells that are critical for BBB *in vitro* modeling and controlling the BBB integrity.

**Table 1:** Main molecular mechanisms and markers of aging-associated alterations in neurovascular unit (NVU).

Aging-associated alterations in NVU	Pathophysiological events in NVU	Molecular markers	Reference
Endothelial dysfunction	Oxidative stress	ROS overproduction, NADPH oxidase activity	Freeman and Keller (2012); Sohrabji et al. (2013)
	Apoptosis	Specific DNA fragmentation, phosphatidylserine exposure	Hoffmann et al. (2001)
	Impairment of tight junctions, adherence junctions, connexin channels	Altered expression of Cx43, Cx40, CLD5, ZO1, JAM	Lei et al. (2013); Elahy et al. (2015)
	Hyperpermeability, hypervascularity	Elevated expression of VEGF, MMP2, MMP9, decrease of TEER, increased permeability for dyes, dextrans, liposomes	Biron et al. (2011); Zhang et al. (2017)
	Reduced proliferation	Decreased expression of Ki67, PCNA	Katsimpardi et al. (2014)
Astroglial dysfunction	Altered metabolism and morphology	Aberrant lactate production	Jiang and Cadenas (2014); Goodall et al. (2018)
	Local glucose hypometabolism	Decreased expression of GLUT4, IRAP, low lactate levels	Mosconi (2013); Camandola and Mattson (2017)
	Neuroinflammation, Inflamm-aging	SASP phenotype (expression of inflammasomes, RAGE, HMGB1, IL-1β, IL-18, IL-33, TNFα, other cytokines and chemokines)	Fu et al. (2014); Chinta et al. (2015)
Pericyte dysfunction	Pericytes loss	Reduced number of pericytes, decreased expression of PDGFR	Bell et al. (2010); Winkler et al. (2010)
Microglial activation and dysfunction	Neuroinflammation, Inflamm-aging	SASP phenotype (overproduction of cytokines, chemokines, ROS), signs of M2 → M1 polarization (expression of Arg1, CD206, and Ym1 vs. IL-1β, TNF-α, IL-6, CD16/32, CD86, CD40, iNOS)	Norden and Godbout (2013); Solano Fonseca et al. (2016)
Neuronal dysfunction	Neuronal loss	Neurogenesis impairments, decreased expression of NeuN, DCX, excessive expression of cell death (apoptosis, autophagy) markers	Sun et al. (2013)
	Local insulin resistance and corresponding metabolic alterations	Altered expression and activity of insulin receptors, GLUT, IRS, PI3K, Akt, GSK3β	Akintola and van Heemst (2015)
	Synaptic dysfunction	Low expression of PSD95, Synaptophysin	Morrison and Baxter (2012); Mostany et al. (2013)

## Conclusion and Future Prospects

As it is clearly seen from above data, the following approaches could be applied in order to induce senescent phenotype in BBB cells *in vitro*: (1) induction of SASP and inflammasome activation; (2) induction of IR; (3) manipulating NAD^+^ levels; (4) induction of DDR associated with γ-H2AX accumulation; and (5) promotion of mitochondrial biogenesis, glycolytic changes and aberrant production of lactate.

It is clear that application of “standard” BBB *in vitro* models to study barrier alterations in the aging brain could be possible if cellular components of the BBB are obtained from the brain of aged animals or animal strains with accelerated aging, i.e., possessing genetically encoded impairments in the signaling of growth hormone/IGF-1, mTOR, sirtuins, suffering from weak antioxidant defense, prone to metabolic alterations, demonstrating prominent inflammatory response or alterations in DNA repair mechanisms, as excellently reviewed in Liao and Kennedy (2014). However, establishment of BBB models using cells isolated from aged animals is usually connected with several technological problems (low rate of cell proliferation, high levels of spontaneous cell death etc.). So, in some cases induction of senescence phenotype should be done in non-aged cells already introduced into the *in vitro* BBB model (or just prior the reconstitution of the desired multi-cellular ensemble). Therefore, careful phenotyping of cells is absolutely needed for getting correct data on BBB/NVU impairments in the aging brain. Development and application of adequate “aging” BBB *in vitro* models would provide further progress in the exploration of brain aging phenomenon, development of novel drug candidates, personification of preventive and therapeutic strategies in patients with age-dependent brain disorders.

## Author Contributions

EO conceived and wrote the manuscript. YK performed the literature review and wrote the manuscript. AM, OL, YP, RO and EV performed the literature review and figures design. VS conceived and edited the manuscript. AS conceived, wrote and edited the manuscript.

## Conflict of Interest Statement

The authors declare that the research was conducted in the absence of any commercial or financial relationships that could be construed as a potential conflict of interest.
